# Improving Electrical Conductivity, Thermal Stability, and Solubility of Polyaniline-Polypyrrole Nanocomposite by Doping with Anionic Spherical Polyelectrolyte Brushes

**DOI:** 10.1186/s11671-015-0997-x

**Published:** 2015-07-25

**Authors:** Na Su

**Affiliations:** School of Printing and Packaging Engineering, Shanghai Publishing and Printing College, Shanghai, 200093 China

**Keywords:** Nanocomposite, Polyaniline-polypyrrole, Anionic spherical polyelectrolyte brushes, Doping, Electrical conductivity, Thermal stability, Solubility

## Abstract

The extent to which anionic spherical polyelectrolyte brushes (ASPB) as dopant improved the performance of polyaniline-polypyrrole (PANI-PPy) nanocomposite was investigated. Different characterization and analytical methods including Fourier transform infrared spectroscopy (FTIR), thermo-gravimetric analysis (TGA), scanning electron microscopy (SEM), and X-ray diffraction (XRD) confirmed that ASPB serving as dopant could improve the comprehensive properties of PANI-PPy nanocomposite. It was different from dopants such as SiO_2_, poly(sodium-*p*-styrenesulfonate) (PSS), and canonic spherical polyelectrolyte brushes (CSPB) which only enhanced the performance of PANI-PPy nanocomposite on one or two sides. The electrical conductivity of (PANI-PPy)/ASPB nanocomposite at room temperature was 8.3 S/cm, which was higher than that of PANI-PPy (2.1 S/cm), (PANI-PPy)/PSS (6.8 S/cm), (PANI-PPy)/SiO_2_ (7.2 S/cm), and (PANI-PPy)/CSPB (2.2 S/cm). Meanwhile, (PANI-PPy)/ASPB nanocomposite possessed enhanced thermal stability and good solubility. In addition, the effects of polymerization temperature, the molecular weight of grafted polyelectrolyte brushes, and storage time on electrical conductivity were discussed.

## Background

During the last several decades, conducting polymers have been the subject of numerous investigations due to their excellent physical and chemical properties originating from their unique π-conjugated system [1–3]. Among the conducting polymers studied, polyaniline (PANI) and polypyrrole (PPy) are of particular interest because of their promising electrical conductivity, high environmental stability, interesting redox properties, magnetoresistance (MR) behaviors, and electrochemical performances [4–10]. In comparison with numerous reports about PANI and PPy, researches on copolymers of aniline and pyrrole are still far from enough. Because the copolymer may overcome the shortcomings of a single π-electron in the homopolymer, obtaining composites with excellent property [11], studies on copolymerization of aniline and pyrrole gradually attract people’s attention. Electrochemical [12, 13] and chemical oxidative polymerization methods [14, 15] are the most common methods used in the synthesis of conducting copolymers. However, the large-scale application of PANI-PPy composite is sometimes limited by the difficulty of insolubility and infusibility of the material which can lead to poor electronic conductivity and mechanical properties. Therefore, the improvement of the comprehensive properties of PANI-PPy nanocomposite is of significance.

To date, most of the published research on this topic has been developed to improve the performance of conducting polymers on certain aspects. Xin et al. [16] prepared poly(aniline-*co*-pyrrole) nanocomposite by chemical oxidation polymerization using iron(III) chloride hexahydrate (FeCl_3_·H_2_O) as an oxidant and sodium dodecylbenzenesulfonate (SDBS) as a surfactant. They found that the nanocomposite had high electrical conductivity by selecting proper conditions for the synthesis process. Li et al. [17] proposed a method to prepare poly(pyrrole-*co*-aniline) nanofibrils using a template by chemical copolymerization technique. It was reported that the length, diameter, and thickness of copolymer nanofibers were controlled by using AAO as a template, and the copolymer nanofibers had good thermal stability. Moreover, as for PPy, enhanced mechanical properties and reduced flammability were obtained by doping with epoxy resin [18]. However, since the properties of conducting polymers are mutually restraining, the improvement of their comprehensive performance is an important and difficult task.

In recent years, conducting polymers doped with polyelectrolyte have achieved outstanding progress [19]. Wu and coworkers [20] developed PPy, which exhibited excellent electrical conductivity and solubility by using different concentrations of water-soluble polystyrene sulfonate (PSS). The reason for this may be that de-doping does not easily happen for doped ions due to the large size of the polyelectrolyte, so the electrical conductivity of conducting polymers is more stable. Meanwhile, the entanglement effect of the long flexible chains of the polyelectrolyte can effectively hinder the growth of copolymer chains, helping to enhance its solubility. It is undoubtedly a good reference for the development of conducting polymers with excellent performance. In addition, in order to improve the thermal stability, magnetoresistance, and processing performance of conductive nanocomposites, besides organic materials just like MWNTs/PANI [21], inorganic materials were used, such as PPy/Fe_3_O_4_ [22, 23], PPy/SiO_2_ [24, 25], and PPy/Co_3_O_4_ [26]. In view of this, highly branched anionic spherical polyelectrolyte brushes (ASPB), consisting of polyelectrolyte chains affixing to the surface of spheres, may be a novel dopant of conducting polymers which can improve the performance of conducting polymers by introducing the brush polymers with certain functional groups.

This paper presented a facile method for the synthesis of (PANI-PPy)/ASPB nanocomposite by chemical oxidative polymerization. On the basis of a previous work [27], the advantage of ASPB serving as dopant in the synthesis of (PANI-PPy)/ASPB nanocomposite was evaluated. Compared to the PANI-PPy nanocomposite doped with PSS, SiO_2_, and canonic spherical polyelectrolyte brushes (CSPB), (PANI-PPy)/ASPB nanocomposite exhibited good performances of electrical conductivity, thermal stability, and solubility. Moreover, since electrical conductivity was an important performance for conducting polymers, the effects of polymerization temperature, the molecular weight of grafted polyelectrolyte brushes, and storage time on electrical conductivity were also studied.

## Methods

### Materials

Aniline and pyrrole (Sinopharm Chemical Reagents Co., Ltd, Shanghai, China) were distilled under reduced pressure before use. Ammonium persulfate (APS, 98 %) was purchased from Sinopharm Chemical Reagents Co., Ltd, Shanghai, China. Other chemicals and solvents including hydrochloric acid (HCl, 36–38 %) and ethanol were analytical reagents and were used without further purification. The ASPB (*D*_z_ ≈ 100 nm, *M*_w_ = 500–2000 g/mol) consisting of modified SiO_2_ cores and PSS brushes were prepared by surface-initiated polymerization [28]. CSPB (*D*_z_ ≈ 100 nm, *M*_w_ = 2000 g/mol) were composed of SiO_2_ cores and poly(diallyldimethylammonium chloride) (p-DMMPAC) brushes.

### Synthesis of (PANI-PPy)/ASPB Composite

**Fig. 1:**
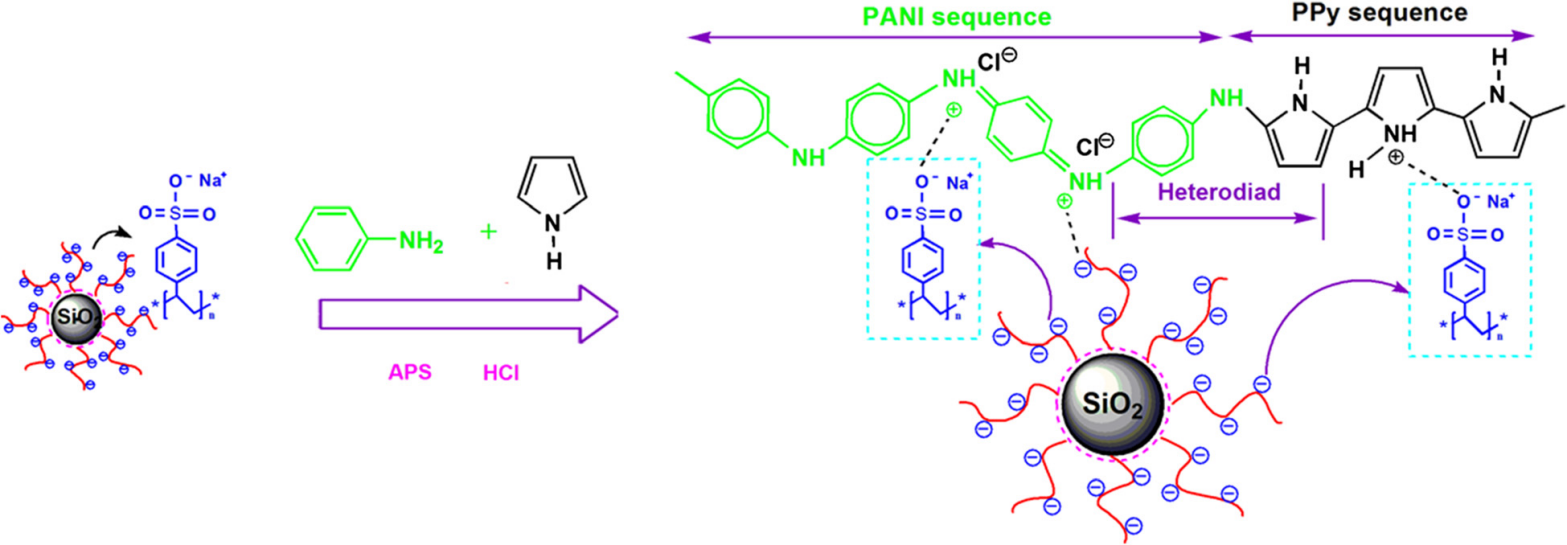
Schematic representation of the synthesis of (PANI-PPy)/ASPB nanocomposite

In this paper, we presented a facile method for the synthesis of (PANI-PPy)/ASPB nanocomposite by chemical oxidative polymerization, and the synthesis process is shown in Fig. 1. In a typical procedure, 92 mg of ASPB was firstly added into 25.2 mL of 2 M HCl (aq) with ultrasonic dispersion for 20 min, and then 1.8 mL of aniline and 1.4 mL of pyrrole were added. After the mixture was cooled to 5 °C and degassed under a flow of N_2_ for 20 min, copolymerization was initiated by dissolving 4.5 g of APS in 12.6 mL of 2 M HCl (aq). After 6 h, the products were collected via filtration and washed with ethanol and distilled water for three times to remove small molecular compounds and unreacted monomers. The resulting products were vacuum dried at 60 °C for 12 h. In order to compare the doping effect of different dopants involving ASPB, PSS, SiO_2_, and CSPB, (PANI-PPy)/PSS, (PANI-PPy)/SiO_2_, (PANI-PPy)/CSPB, and PANI-PPy nanocomposites were synthesized.

### Characterization

Fourier transform infrared spectroscopy (FTIR) was obtained using a Nicolet AVATAR 360FT spectrometer (USA). The chemical composition of (PANI-PPy)/ASPB nanocomposite was inspected by energy-dispersive X-ray diffraction (EDX) spectroscopy attached to a scanning electron microscope (SEM) which was used to investigate the morphology of samples. It was recorded on a Quanta 200 microscope (FEI, Netherlands) operated at 30 kV. X-ray diffraction (XRD) measurements were performed on a Shimadzu “XRD-6000” instrument (Japan) operating at a voltage of 40 kV and a current of 40 mA with CuK_α_ radiation, *λ* = 1.54060 Å. The samples were measured in a continuous scan mode at 5°–50° (2*θ*) with a scanning rate of 5°/min. Thermo-gravimetric analysis (TGA) was carried out on a SETSYS-1750 instrument at a heating rate of 10 °C/min under nitrogen atmosphere.

### Measurements

The electrical conductivity of the samples was measured in a four-point probe apparatus (RTS-4, China) at room temperature. The samples were firstly pressed into a circular tablet with *D* = 13 mm at 20 MPa, and the measurement of the thickness *W* of each tablet was then performed. In order to calculate the source current *I* according to formula (1) [29], *F*(*W*/*S*) and *F*(*D*/*S*) (*S* = 1) which denoted the width correction coefficient and the diameter correction coefficient, respectively, were looked up from a table. The resistivity *ρ* at room temperature could be obtained from the four-point probe apparatus. Since conductivity (*σ*; S/cm) = 1/*ρ*, the electrical conductivity of the samples was calculated.

1 $$ I=F\left(W/S\right)\times F\left(D/S\right)\times W\times 0.1 $$

The solubility of the samples can be reflected by the conductivity of the saturated solution (*T* = 25 °C, pH = 6). It is measured with a DDS-12A digital conductivity meter (Hubei Provincial Institute of Measurement and Testing). The specific process is as follows: 10 mg of samples was dissolved in 4 mL of ethanol with stirring and heating to boiling, then the supernatant and soluble impurities were removed. The process was repeated twice. After 10 mL of ethanol was added and heated to boiling for the purpose of fully dissolving the samples, the samples were placed in a bath at constant temperature for 20 min to precipitate the solid. To avoid the effect of the solid particles suspended in the electrode on experimental results, 3 or 4 mL of supernatant was added into the beaker. Additionally, the conductivity of the saturated solution of ethanol used as reference was required to be measured.

## Results and Discussion

### FTIR Spectra

**Fig. 2:**
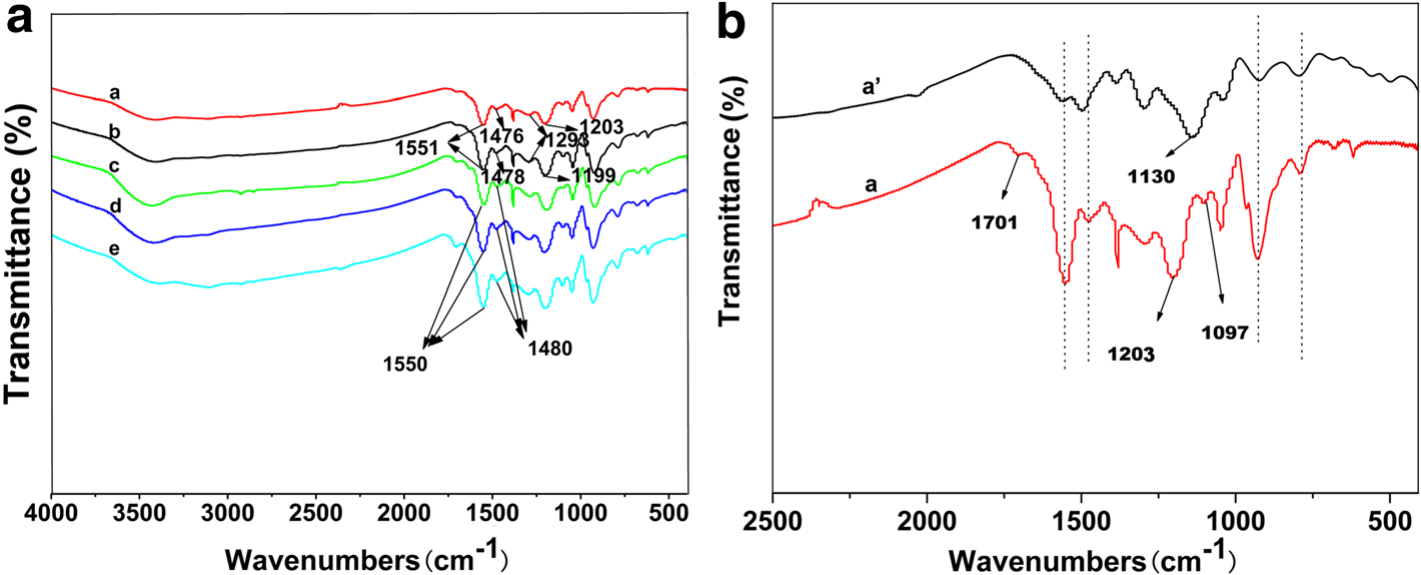
**a** FTIR spectra of (*a*) PANI-PPy, (*b*) (PANI-PPy)/ASPB, (*c*) (PANI-PPy)/PSS, (*d*) (PANI-PPy)/CSPB, and (*e*) (PANI-PPy)/SiO_2_ nanocomposites. **b** FTIR spectra of (*a*) PANI-PPy nanocomposites and (*a’*) the blends of the two homopolymers

FTIR spectroscopy is used to study the chemical bonding of samples (see Fig. 2). The characteristic peaks of PANI-PPy, represented by the absorption bands at 1551 and 1476 cm^−1^, can be attributed to the stretching vibration of the quinonoid and benzenoid rings. The peaks at 1293 and 1203 cm^−1^ are assigned to C–N and C=N stretching vibrations, respectively, which are consistent with the literature [30]. While adding a variety of different dopants, no new peaks appear. However, the ratio of the integrated absorption areas at approximately 1476 and 1551 cm^−1^ (A1476/A1551) for the composites is different, indicating that they have different conjugation lengths. The higher ratio represents the longer conjugation length [31]. The conjugation length follows the order (PANI-PPy)/ASPB > (PANI-PPy)/SiO_2_ > (PANI-PPy)/PSS > (PANI-PPy)/CSPB, which is also the order of the electrical conductivity of nanocomposites.

In order to demonstrate that the conducting composites are composed of copolymers of aniline and pyrrole, instead of a simple blend of the homopolymers, the contrast FTIR spectra of the copolymers and blends are also shown in Fig. 2b. As can be seen from the figure, most characteristic absorption peaks for the blends also appear in the spectrum of the copolymers. However, compared with the blends which have only one C–N stretching vibration at 1130 cm^−1^ (Fig. 2b (a’)), two C–N stretching vibrations (1203 and 1097 cm^−1^) and a carbonyl group (1701 cm^−1^) are displayed (Fig. 2b (a)), which is consistent with the literature [32].

### XRD Patterns

**Fig. 3:**
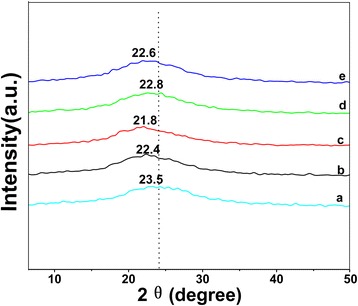
XRD patterns of (*a*) PANI-PPy, (*b*) (PANI-PPy)/ASPB, (*c*) (PANI-PPy)/SiO_2_, (*d*) (PANI-PPy)/CSPB, and (*e*) (PANI-PPy)/PSS nanocomposite

The crystallographic structure of the samples is probed by X-ray diffraction. As shown in Fig. 3, the broader peak in the XRD pattern of PANI-PPy nanocomposite is observed at 2*θ* = 23.5°, implying an amorphous structure [31]. For the (PANI-PPy)/ASPB nanocomposite, the characteristic peak has shifted to 2*θ* = 22.4°. Similarly, for (PANI-PPy)/ASPB, (PANI-PPy)/SiO_2_, (PANI-PPy)/PSS, and (PANI-PPy)/CSPB nanocomposites, only a broad peak presents in their XRD spectra, but these peak positions have shifted to 2*θ* = 22.4°, 21.8°, 22.6°, and 22.8°, respectively. Accordingly, the interplanar distances (*d*) corresponding to these diffraction peaks are 0.391, 0.402, 0.389, and 0.385 nm separately calculated by the Prague formula (2*d*sin*θ* = *nλ*) [33]. Compared to PANI-PPy (0.373 nm), the interplanar distance can be increased by doping. The reason for this may be that the addition of dopants changes the original lattice structure of the conductive composite, and the morphological changes are expected, which will be observed in SEM images.

### Electrical Conductivity

Electrical conductivity is determined using a RTS-4 four-point probe which is a simple apparatus for measuring the resistivity of conductive composites. Results indicate that the room-temperature electrical conductivities of PANI-PPy, (PANI-PPy)/PSS, (PANI-PPy)/SiO_2_, and (PANI-PPy)/CSPB nanocomposites are 2.1, 6.8, 7.2, and 2.2 S/cm, respectively, while (PANI-PPy)/ASPB nanocomposite shows a high value of electrical conductivity (8.3 S/cm). The increase in magnitude of the electrical conductivity is consistent with the FTIR results.

### Thermal Stability

**Fig. 4:**
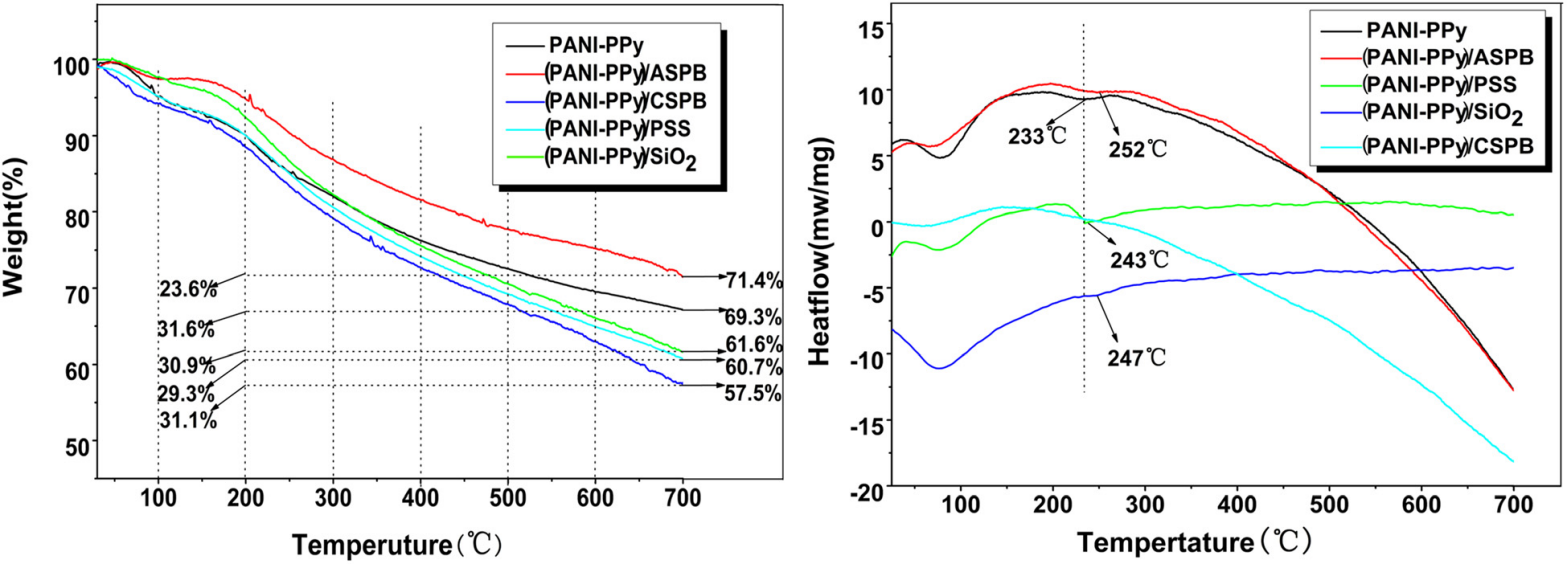
Effect of polymerization temperature and the molecular weight of grafted polyelectrolyte brushes on electrical conductivity

Figure 4 displays the thermo-gravimetric analysis of PANI-PPy, (PANI-PPy)/ASPB, (PANI-PPy)/CSPB, (PANI-PPy)/SiO_2_, and (PANI-PPy)/PSS nanocomposites under N_2_ atmospheres at 60 % RH. As observed from the TGA curve of PANI-PPy, the decreased water content is because of the hydrophobic property of PPy and PANI [34]. A large amount of mass loss of copolymers begins at 200 °C, and the mass loss of copolymers in the temperature range of 200–700 °C is 31.6 %, which is mainly due to the degradation of the polymer [35]. When doped with ASPB, the mass loss of (PANI-PPy)/ASPB nanocomposite in the same temperature range was 23.6 % with a 7 % decrease, indicating that the addition of ASPB can improve the thermal stability of the conductive composite. It may be the reason that the generated direction of polymer chains for polypyrrole and polyaniline is promoted, which will be proved by the SEM images. It also can be seen from the TGA curves of the PANI-PPy nanocomposite doped with CSPB, PSS, and SiO_2_ that the mass losses of (PANI-PPy)/ASPB, (PANI-PPy)/CSPB, (PANI-PPy)/PSS, and (PANI-PPy)/SiO_2_ at 200–700 °C were 23.6, 30.9, 31.1, and 29.3 %, respectively, which are lower than that of PANI-PPy (31.6 %). Results show that the addition of PSS and CSPB can improve the thermal stability of PANI-PPy nanocomposite, but the effect is not obvious. The DSC curves of PANI-PPy nanocomposite with different dopants are also shown in Fig. 4. Compared with PANI-PPy nanocomposite, nearly 20 °C (from 233 to 252 °C) decomposition temperature is increased by the addition of ASPB.Since (PANI-PPy)/ASPB nanocomposite also has a higher decomposition temperature than (PANI-PPy)/CSPB, (PANI-PPy)/PSS, and (PANI-PPy)/SiO_2_, better thermal stability is obtained.

### Solubility

**Fig. 5:**
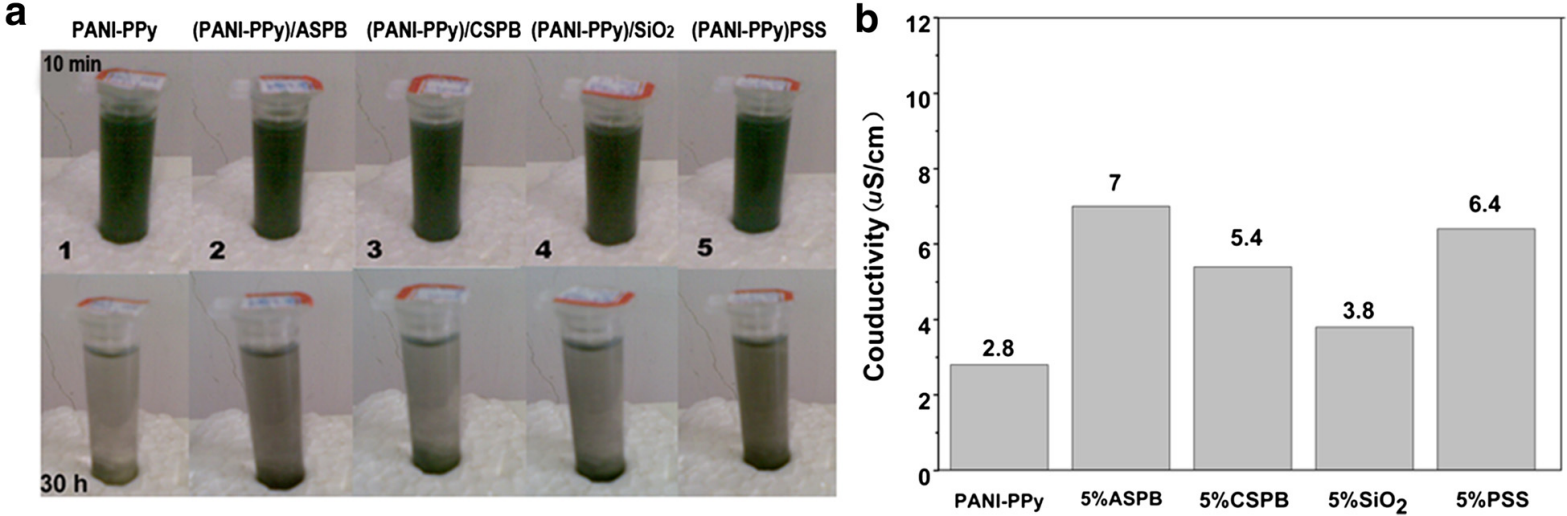
**a** Qualitative and **b** quantitative analysis of the conductivity of saturated solution of PANI-PPy nanocomposite with different dopants (*T* = 25 °C, pH = 6)

Figure 5 represents the solubility of the samples which is reflected by the conductivity of the saturated solution (*T* = 25 °C, pH = 6). As shown in Fig. 5a, the PANI-PPy nanocomposite with different dopants (0.01 g) are dissolved in ethanol (4 mL) with ultrasonic dispersion for 10 min and then placed for 30 h. If the conductive nanocomposite has good solubility, its solution should be green. For the PANI-PPy nanocomposite doped with 5 wt% SiO_2_, PSS, CSPB, and ASPB, different from the (PANI-PPy)/SiO_2_ and (PANI-PPy)/CSPB nanocomposites whose solutions are clear, the solutions show a dark green color for (PANI-PPy)/PSS and (PANI-PPy)/ASPB nanocomposites, indicating that the solubility for these two composites is good. The main reason is that the resulting copolymer chains become short and small due to the flexible long chains of PSS, which makes the nanocomposite well dissolved in the solvent. In addition, the entry of PSS in the PANI-PPy chains may make copolymer chains grow in a direction such that the crosslinking degree is minimal. The addition of ASPB can improve the solubility of conductive nanocomposites which is mainly because of the grafting of PSS brushes. The crystallinity of conductive composites increases with the addition of SiO_2_, thus leading to a larger grain size sample, and a poor solubility is obtained. This result is consistent with the results of XRD. The quantitative analysis method is also shown in Fig. 5b. After testing the conductivity of the saturated solution of ethanol (0.2 μS/cm), the conductivities of the saturated solution of PANI-PPy, (PANI-PPy)/ASPB, (PANI-PPy)/CSPB, (PANI-PPy)/SiO_2_, and (PANI-PPy)/PSS are 2.8, 7, 5.4, 3.8, and 6.4 μS/cm, respectively. Therefore, the contributions of different dopants on the solubility of conductive composites follow the order ASPB > PSS > CSPB > SiO_2_.

### Evaluation of the Effects of Dopants

In summary, a variety of characterization methods prove that the effects of dopant on PANI-PPy nanocomposite are different. The performances of conductivity, solubility, and thermal stability for conductive nanocomposites when the amount of dopant is 5 wt% are shown in Table 1.

**Table 1:** Effect of dopant species on the performance of conductive nanocomposites

Performance	Sample				
	PANI-PPy	(PANI-PPy)/ASPB	(PANI-PPy)/SiO_2_	(PANI-PPy)/PSS	(PANI-PPy)/CSPB
Electrical conductivity (S/cm)	2.1	8.3	7.2	6.8	2.2
Conductivity of saturated solution (μS/cm)	2.8	7	3.8	6.4	5.4
Exothermic peak (°C)	233	252	247	243	Unconspicuous

It can be observed from the table that the contributions of each dopant on electrical conductivity, solubility, and thermal stability of PANI-PPy nanocomposite follow the order ASPB > SiO_2_ > PSS > CSPB, ASPB > PSS > CSPB > SiO_2_, and ASPB > SiO_2_ > PSS > CSPB, respectively. When doped with CSPB, the performances of conductive nanocomposites are not significantly improved except their solubility, indicating CSPB are not suitable as the dopant of conducting polymers. It may be due to the repulsive interaction between their cationic charge of brush layers and radical cation of conducting copolymers, so that CSPB do not enter into the conducting copolymer chains. For (PANI-PPy)/SiO_2_ nanocomposite, the addition of SiO_2_ may increase the structure regularity of conducting polymers, so the electrical conductivity and thermal stability of conductive composites improve. However, the insolubility of SiO_2_ in most solvents also makes it powerless in improving the solubility of conducting copolymers. Furthermore, the main advantage of PSS is that it can increase the solubility of PANI-PPy nanocomposite. On the one hand, its flexible polymer chains in the copolymer system hinder the growth of the copolymer chain, making it smaller and shorter. On the other hand, the hydrophilicity of PSS also promotes the water solubility of conducting copolymers. But studies have shown that it has no obvious effect on improving the thermal stability. For ASPB consisting of a SiO_2_ core and PSS brushes, just a combination of the advantages of SiO_2_ and PSS is conducted. The thermal stability and solubility of copolymers are enhanced by SiO_2_ particles and PSS chains, respectively. Therefore, ASPB are an excellent dopant which can improve the comprehensive performances of PANI-PPy nanocomposite.

### Morphology

**Fig. 6:**
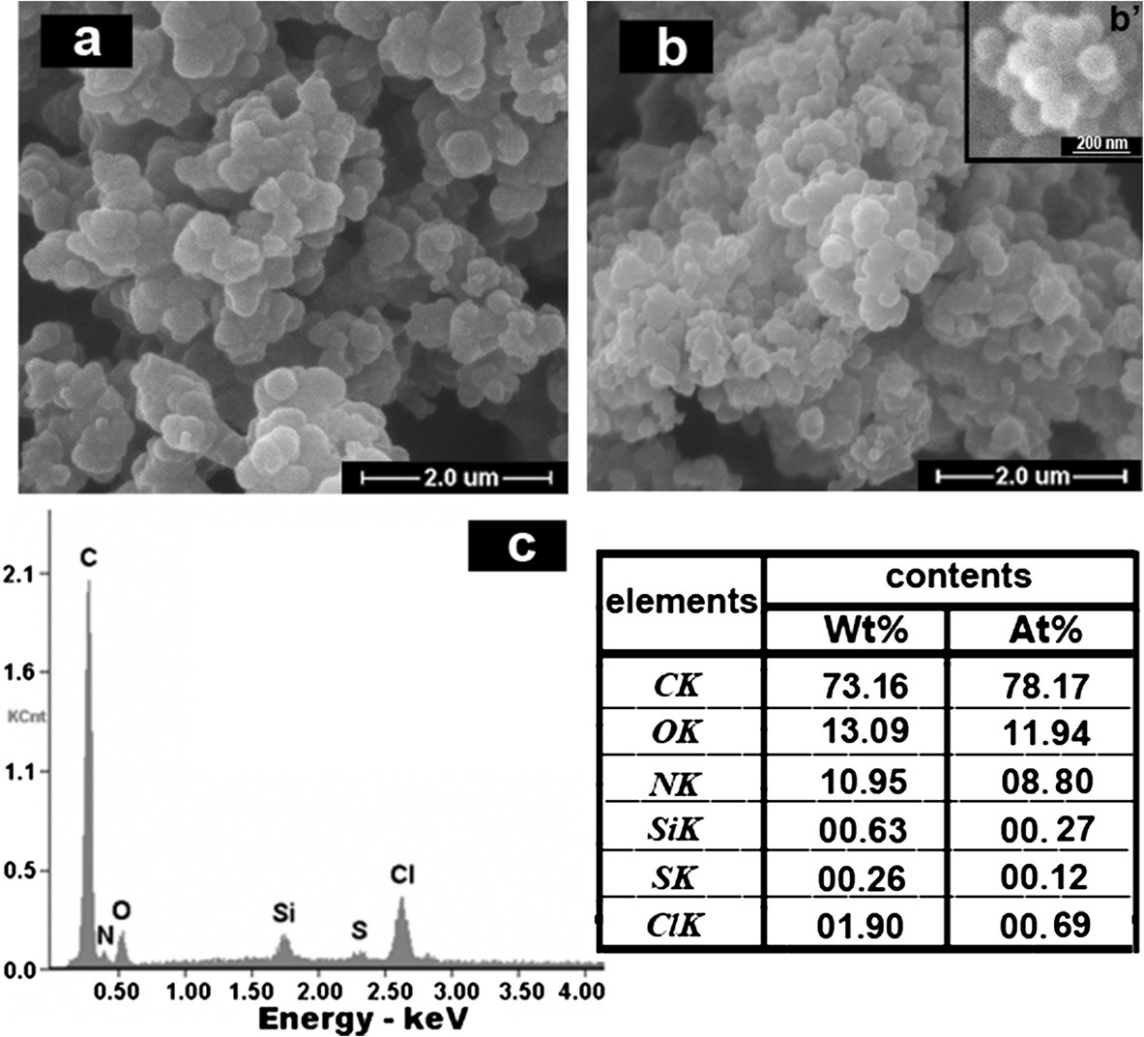
SEM images of **a** PANI-PPy, **b** (PANI-PPy)/ASPB composite, and (*b’*) ASPB. **c** EDX analysis of (PANI-PPy)/ASPB nanocomposite

In order to explore the doping mechanism of ASPB, the morphologies of PANI-PPy (a), (PANI-PPy)/ASPB nanocomposite (b), and ASPB (b’) are studied using SEM images (see Fig. 6), and the chemical composition of (PANI-PPy)/ASPB nanocomposite is determined by the simultaneous EDX spectroscopy (c). As shown in Fig. 6a, PANI-PPy displays a closely stacked granular structure, while the micrograph of (PANI-PPy)/ASPB nanocomposite (Fig. 6b) shows a spherical-like structure. It may be because the addition of ASPB with uniform spherical structure (Fig. 6b (b’)) provides the space factors for copolymer orderly growth, which is consistent with the XRD and TGA results. ASPB serve as template for the synthesis of (PANI-PPy)/ASPB nanocomposite. The EDX analysis shows that the signals corresponding to silicon and sulfur appear on the spectrum, suggesting that the doping of ASPB in the PANI-PPy matrix has occurred.

### Doping Mechanism

**Fig. 7:**
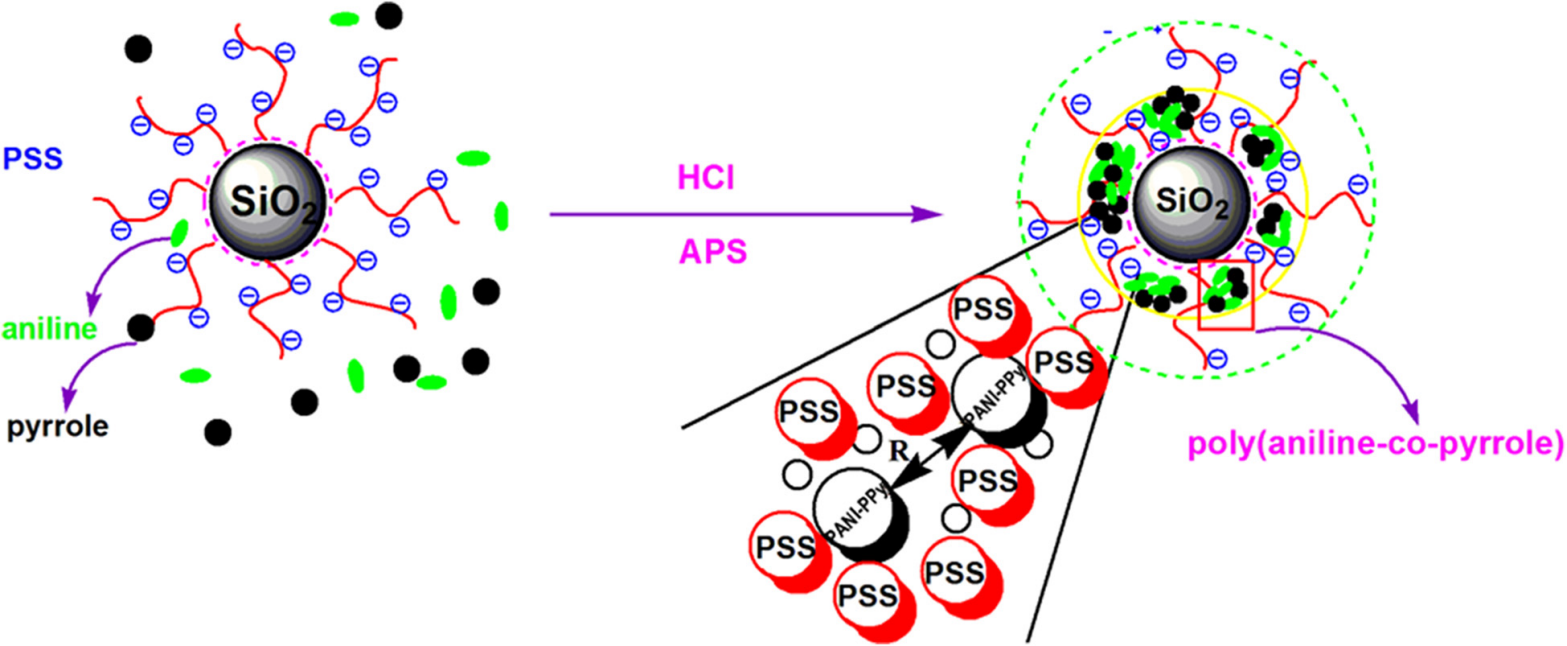
Doping mechanism of ASPB

Figure 7 describes the doping mechanism and specific distribution in the ASPB within a charged reagent. The copolymerization reaction of aniline and pyrrole monomers in the PSS chains happens. *R* denotes the distance between the two copolymer chains. The amount of negative charge per unit length in PSS chains is much more than the amount of positive charge per unit length in PANI-PPy chains, so part of SO^3−^ is balanced by Na^+^. Due to the effects of density gradient formed by densely grafted PSS chains, copolymerization reaction occurs preferentially in high-concentration aniline and pyrrole monomers [36], which are located at the cores of ASPB. According to the doping mechanism of protonic acid, the copolymerization of aniline and pyrrole monomers forms a polymeric cation, which raises the function of multivalent counter ions in brush layers. Therefore, the complexation between PANI-PPy and PSS is irreversible. As a result, the copolymerization of aniline and pyrrole monomers is fixed in the brush layers, and ASPB play a role as template in the synthesis of (PANI-PPy)/ASPB nanocomposite.

### Effect of Polymerization Temperature and the Molecular Weight of Grafted Polyelectrolyte Brushes on Electrical Conductivity

**Fig. 8:**
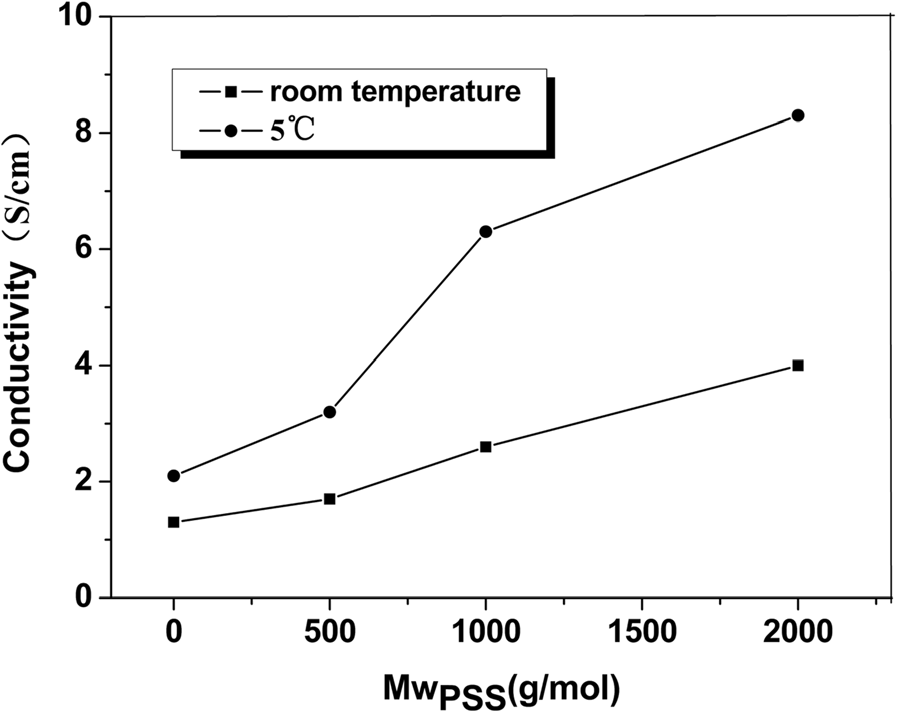
Thermal gravimetric analysis of PANI-PPy, (PANI-PPy)/ASPB, (PANI-PPy)/SiO_2_, (PANI-PPy)/CSPB, and (PANI-PPy)/PSS nanocomposites

The influences of polymerization reaction temperature and the molecular weight of PSS brushes on electrical conductivity are investigated, with 5 wt% ASPB used and 6 h reaction time. As shown in Fig. 8, the electrical conductivity of PANI-PPy composite at room temperature is lower than that at low temperatures (5 °C). The reason for this is that the polymerization process is slow at low temperatures, the content of defect structure in the molecular structure of copolymers decreases along with orderly improvement, which helps to increase the electrical conductivity. While at high reaction temperature, the formation of increased defects in the copolymer makes the surface morphology rough, thereby affecting electrical conductivity [37]. In addition, when other conditions remain the same, ASPB with different molecular weights of grafted brushes (500, 1000, and 2000 g/mol) are selected for the doping experiment, and the results are also shown in Fig. 8. The electrical conductivities of (PANI-PPy)/ASPB nanocomposites increase with the increase of the molecular weight of grafted polyelectrolyte brushes. The reason for this may be that the long chains of the polyelectrolyte make pyrrole and aniline monomers grow better in the ASPB system. The crosslinking degree of conducting copolymer chains reduces, resulting in generating a polymer chain with more extended and extended conjugation length, so the electrical conductivity increases [19].

### Effect of Storage Time on Electrical Conductivity

**Fig. 9:**
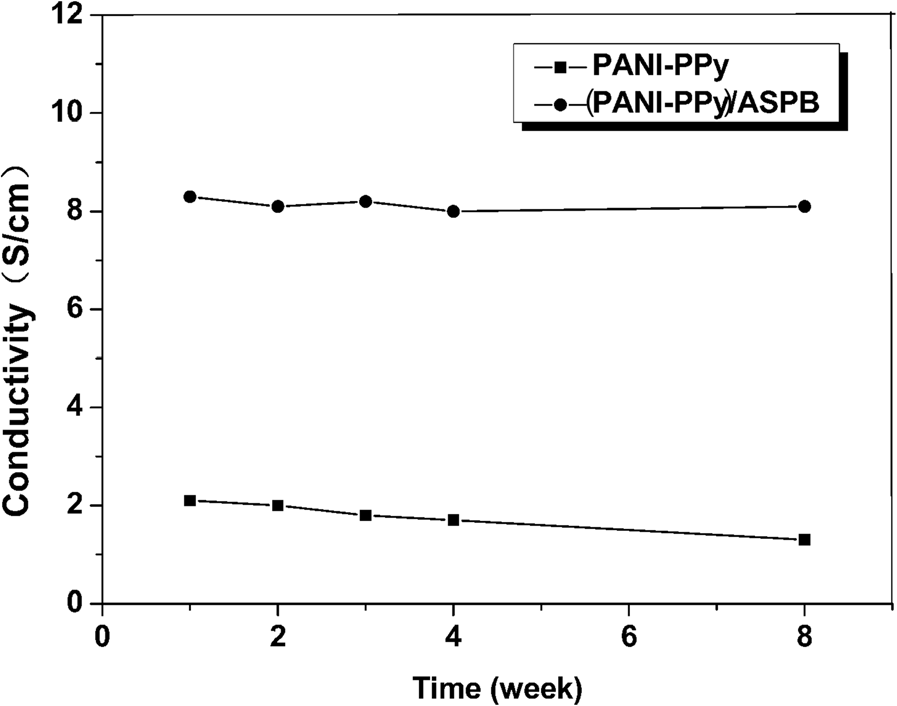
Effect of storage time on the electrical conductivity

The PANI-PPy nanocomposite doped with proton acid and ASPB is placed in the air for a period of time at room temperature to study the stability of electrical conductivity. As shown in Fig. 9, the electrical conductivity of PANI-PPy nanocomposite doped with ASPB is almost unchanged after a few months, while the electrical conductivity of the small ion-doped nanocomposite declines. It is mainly because that the de-doping phenomenon is difficult to happen when macromolecular doping is conducted, and the structure of copolymer chains is more stable.

## Conclusions

This paper proposes a simple and effective method to improve the comprehensive performance of conductive composites. By doping the ASPB, the electrical conductivity, thermal stability, and solubility of PANI-PPy composite are enhanced. The electrical conductivity of (PANI-PPy)/ASPB nanocomposite is 8.3 S/cm, which is higher than that of (PANI-PPy)/CSPB (2.2 S/cm), (PANI-PPy)/PSS (6.8 S/cm), and (PANI-PPy)/SiO_2_ (7.2 S/cm). Furthermore, the influences of polymerization temperature, the molecular weight of grafted polyelectrolyte brushes, and storage time on the electrical conductivity of (PANI-PPy)/ASPB nanocomposite are investigated. Results show that the long grafted chains and low reaction temperature help to improve the electrical conductivity of conductive composites.
